# Central nervous system Toll-like receptor expression in response to Theiler's murine encephalomyelitis virus-induced demyelination disease in resistant and susceptible mouse strains

**DOI:** 10.1186/1743-422X-5-154

**Published:** 2008-12-18

**Authors:** Nicolas P Turrin

**Affiliations:** 1 Molecular Endocrinology & Oncology Research Centre (CHUQ), 2705 Laurier Blvd., Québec City, Qc., G1V 4G2, Canada

## Abstract

**Background:**

In immunopathological diseases, such as multiple sclerosis (MS), genetic and environmental factors that contribute to the initiation and progression of the disease are often discussed. The Theiler murine encephalomyelitis virus-induced demyelination disease (TMEV-IDD) model used to study MS reflects this: genetically susceptible mice infected intra-cerebrally with TMEV develop a chronic demyelination disease. TMEV-IDD can be induced in resistant mouse strains by inducing innate immunity with lipopolysaccharide (LPS). Interestingly, Toll-like receptor 4 (TLR4) is the cognate receptor for LPS and its activation can induces up-regulation of other TLRs, such as TLR7 (the receptor for TMEV) and 9, known to be involved in autoimmunity. Up-regulation of TLRs could be involved in precipitating an autoimmune susceptible state. Consequently, we looked at TLR expression in the susceptible (SJL/J) and resistant (C57BL/6) strains of mice infected with TMEV. The resistant mice were induced to develop TMEV-IDD by two LPS injections following TMEV infection.

**Results:**

Both strains were found to up-regulate multiple TLRs (TLR2, 7 and 9) following the TMEV infection. Expression of these TLRs and of viral mRNA was significantly greater in infected SJL/J mice. The susceptible SJL/J mice showed up-regulation of TLR3, 6 and 8, which was not seen in C57BL/6 mice.

**Conclusion:**

Expression of TLRs by susceptible mice and the up-regulation of the TLRs in resistant mice could participate in priming the mice toward an autoimmune state and develop TMEV-IDD. This could have implications on therapies that target TLRs to prevent the emergence of conditions such as MS in patients at risk for the disease.

## Background

It is now widely accepted that the central nervous system (CNS) contains its own immune system to protect it from infection and to repair injury. At the core of this response are the microglia, which play the role of macrophages in the CNS. Through the Toll-like Receptor (TLR) family of receptors, microglia are able to recognize pathogen-associated molecular patterns, thereby initiating innate immunity in the brain. Once activated, innate immunity can mobilize the microglia, as well as invading macrophages from the periphery, to clear pathogens and debris from the CNS. This response also serves as a bridge to orchestrate adaptive immunity if needed. Nevertheless, an immune response in the CNS can have drastic consequences if left unchecked. For example, by having other immune cells invade the CNS, including T-cells, the risk of developing an immune response against self-antigens has to be considered. Such a case is believed to exist in multiple sclerosis (MS), whereby antibodies are generated against myelin proteins, leading to the destruction of the myelin sheath of neurons and the associated neurological dysfunctions.

The concept of autoimmunity in MS has been extensively explored by various animal models. One of the most common is experimental autoimmune encephalomyelitis (EAE), where an adjuvant is given with myelin protein in order to incite a self-response to myelin in the rodent. Another model that is gaining importance is the Theiler murine encephalomyelitis virus-induced demyelination disease (TMEV-IDD) model of MS. In this model, susceptible (SJL/J, for example) mice are infected intracerebrally with the TMEV and, following encephalitis and a latency phase, they develop the chronic on-going TMEV-IDD, with recurring demyelination and associated motor deficits. In resistant mouse stains, the initial encephalitis occurs, but no TMEV-IDD persists. However, if innate immunity is stimulated in resistant mice through the systemic administration of lipopolysaccharide (LPS), a ligand for the TLR4 receptor, susceptibility and clinical symptoms associated with TMEV-IDD are increased[1]. Interestingly, the single stranded RNA genome of TMEV is believed to bind to TLR7 and its double stranded replication intermediate to TLR3 located the endosomes and lysosomes of host cells[2]. Since LPS is known to up-regulate multiple TLRs in the CNS's microglia and invading macrophage[3,4], this could be at the source of the enhanced susceptibility of LPS-treated mice infected with TMEV. Indeed, TLR4-dependent activation of innate immunity has been suggested to be involved in infection-induced immune diseases[5]. In addition to the recent reports that TLRs can bind endogenous molecules[6], it becomes imperative to characterize the TLR profile in the CNS of TMEV-IDD susceptible and resistant strands of mice following infection with the virus.

In this study, the TLR mRNA expression profiles of the TMEV-IDD susceptible SJL/J and resistant C57BL/6 mice were determined by *in situ *hybridization a month following TMEV infection. The C57BL/6 mice were treated with LPS in order to promote the chronic infection and demyelination state. By comparing the TLR expression profile of TMEV-IDD resistant and susceptible mouse strains, insight about potential TLRs involved in the pathogenesis of TMEV-IDD could be revealed.

## Methods

### C57BL/6 and SJL/J mice

Adult (8 week old) male (25–35 g) C57BL/6 (n = 30) and SJL/J (n = 15) mice were originally purchased from Charles Rivers Canada, St. Constant, QC, Canada. The C57BL/6 mice are known to be resistant to the TMEV, while the SJL/J mice are susceptible to chronic TMEV infection[7]. All animals were acclimated to standard laboratory conditions (14-h light, 10-h dark cycle; lights on at 06:00 and off at 20:00 h) with free access to rodent chow and water for a week. All protocols were conducted according to the Canadian Council on Animal Care guidelines, as administered by the Laval University Animal Welfare Committee.

### Theiler's Murine encephalomyelitis virus preparation and intracerebral injection

**Figure 1:**
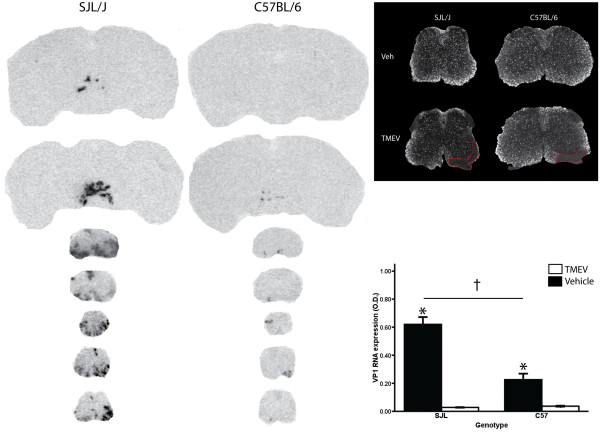
**Theiler's murine encephalomyelitis virus infection can be established in resistant C57BL/6 mice following lipopolysaccharide intraperitoneal injections**. Photomicrographs of X-ray films from *in situ *hybridization (ISH) signals for viral protein 1 (VP1) RNA expression in the brain and spinal cord of SJL/J and C57BL/6 mice one month following Theiler's murine encephalomyelitis virus (TMEV) infection (intracerebral, 2 × 10^5 ^p.f.u.). In order to render the C57BL/6 susceptible to TMEV, LPS injections (20 μg, i.p., Sigma Aldrich) were given immediately following the TMEV injection and +5 days post-infection. A majority of the C57BL/6 mice receiving the (lipopolysaccharide) LPS (6/10) developed a TMEV infection (as shown by the expression of VP1, above) while one mouse (1/10) not receiving the LPS developed a lasting infection (not shown). Inset (top): dark field photomicrography of proteolipid protein (PLP) mRNA expression following ISH. Hybridized slides were dipped into NTB emulsion milk (Kodak). All SJL/J and C57BL/6 mice that developed an infection showed demyelination, as exemplified by the absence of PLP expression in white mater areas of the spinal cord (dashed line area). Bottom panel shows quantitative analysis of VP1 optical density (O.D.) in representative spinal cord sections. Although comparisons revealed that VP1 expression was significantly lower in infected C57BL/6 *versus *SJL/J mice, VP1 expression in C57BL/6 was still significantly higher in the TMEV infected *versus *vehicle saline group. Data presented as mean ± SEM. †: significant SJL/J *vs *C57/BL6 pair wise comparison within TMEV treatment, Bonferonni corrected *t*-test p < 0.05; *: significant TMEV *vs *Vehicle within strain pair wise comparison Bonferonni corrected *t*-test p < 0.05.

The plasmid containing the Daniel strain of TMEV was a generous gift from T. Michiels (Université Catholique de Louvain, Brussels, Belgium). The plasmid was replicated, purified and transferred by electroporesis into adherent BHK-21 cells. Viral concentration was determined as plaque forming units (pfu) on cultures of the same strain. The mice were anesthetized and injected intracerebrally with 2 × 10^5 ^pfu of TMEV or vehicle saline with the use of a sterile syringe fitted with a William's collar. The injection is made halfway between the back of the eye and the ear, at a 45 degree angle from the top of the skull. Infection was confirmed by *in situ *hybridization for RNA coding for viral protein (see below). To increase sensitivity of the C57BL/6 to the TMEV, the protocol of Kim and colleagues[1] was followed: two injections of LPS (20 μg/0.1 ml, i.p., Sigma Aldrich) in sterile saline were given day 0 and +5 post-infection with the virus. Mice were sacrificed 30 days following the infection with the TMEV, when the infection and demyelination is thought to be well established (see Fig. 1).

To collect the brain and spinal cord tissues, the mice were deeply anesthetized via an i.p. injection of a mixture of ketamine hydrochloride and xylazine, and then rapidly perfused transcardially with 0.9% saline, followed by 4% paraformaldehyde/3.8% Borax in sodium phosphate buffer (pH 9 at 4°C). The brains and spinal cords were rapidly removed, post-fixed overnight and then placed in a solution containing 10% sucrose diluted in 4% paraformaldehyde/3.8% Borax buffer (pH 9) overnight at 4°C. The brains were mounted on a microtome (Reichert-Jung, Cambridge Instruments Company, Deerfield, IL, USA), frozen with dry ice, and cut into 25 μm coronal sections from the olfactory bulb to the end of the medulla. Representative 2 mm segments from the cervical, thoracic and lombosacral regions of the spinal cord were collected in 20 μm thick coronal sections. The slices were collected in a cold cryoprotectant solution (0.05 M sodium phosphate buffer, pH 7.3, 30% ethylene glycol, 20% glycerol) and stored at -20°C.

### Viral RNA and TLR mRNA expression and demyelination analysis using in situ hybridization

In order to detect the TMEV, a cDNA probe was generated against the VP1 viral protein coding region of the TMEV. Furthermore, the expression of TLRs binding specific viral (TLR3, TLR7, and TLR8) and bacterial (TLR2, TLR4, and TLR6) elements (TLR9 recognizes unmethylated CpG elements in both viral and bacterial DNA) was done using cDNA probes against their respective mRNA. All cDNA probes were generated by PCR amplification. *In situ *hybridization (ISH) was performed on every 12^th ^section of the collected brain and spinal cord tissue using ^35^S-labeled cRNA probes as described previously [8-10].

In order to visualize the demyelination done by the TMEV, ISH was done on the tissue for PLP as described above. The lack of PLP expression is a reliable measure of demyelination in other models of CNS damage used previously[11].

### Co-localization of TMEV RNA within neuronal cell types using a combination of immunocytochemistry with in situ hybridization

Immunocytochemistry (IC) was combined with the ISH protocol to determine whether viral RNA was expressed in microglia, astrocytes, oligodendrocytes, neurons, and T-cells in the infected mice. Immunocytochemistry against anti-ionized calcium binding adapter molecule 1 (*iba1*, Wako Chemicals, Richmond VA, labeling infiltrating macrophages and microglia), glial fibrillary acidic protein (*GFAP*, Chemicon International, Temicula, CA, labeling astrocytes), Neuronal Nuclei (*NeuN*, Chemicon International, labeling neurons), carbonic anhydrase II (*CAII*, a generous gift from Dr. S. Ghandour, Université Louis Pasteur, Strasbourg, France, labeling oligodendrocytes) and T-cell receptor alpha beta (*TCRαβ*, Cedar Lane Laboratories, Burlington, ON, Canada, labeling most T-cells) was followed by ISH (TLR2 mRNA) as described previously[12].

### Data analysis

The relative intensity of mRNA signals was measured on Biomax MR X-ray films (Kodak, Rochester, NY, USA). Transmittance values (referred to in this study as O.D.) of positive hybridization signal were measured under a Northern Light desktop illuminator (Imaging Research, Ste-Catherine's, ON, Canada) using a Sony camera video system attached to a MicroNikkor 55-mm Vivitar extension tube set for a Nikon lens and coupled to a Dimension GX270 personal computer (Dell Computers, North York, ON, Canada)) and ImageJ software (version 1.23, W. Rasband, National Institute of Health, Bethesda, MD, USA). O.D. for each pixel was calculated using a known standard of intensity and distance measurements from a logarithmic specter adapted from BioImage Visage 110 s (Millipore, Ann Arbor, MI, USA). Eight spinal cord sections from experimental animals were digitized and subjected to densitometric analysis, yielding average peak O.D.'s. The O.D. for each section was corrected for the average background signal on the film. To standardize the sampling procedure, the 8 sections showing the strongest signal were analyzed and averaged.

### Statistical analysis

Data were compiled and the statistical analysis was performed using SigmaStat (Systat, San Jose, CA) software (version 3.5), with TMEV vs. saline treatment, and SJL/J vs. C57BL/6 strain differences as independent variables. Between-group differences of RNA expression density were analyzed using analysis of variance (ANOVA). All pair wise multiple comparison procedures were conducted using Bonferroni corrected *t*-tests. An alpha < 0.05 was considered significant.

## Results

### TMEV infection of susceptible SJL/J versus resistant C57BL/6 mice

**Figure 2:**
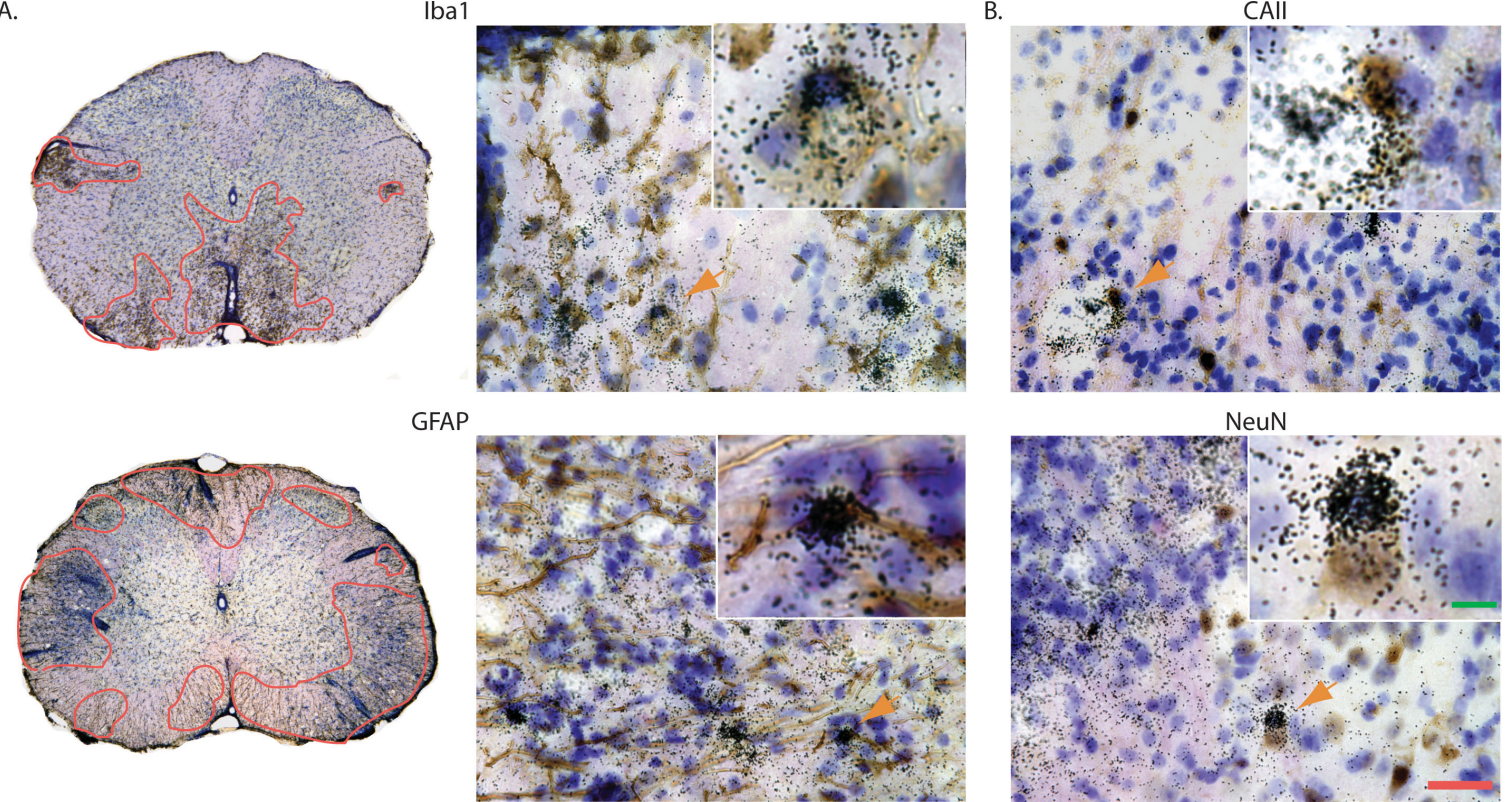
**Viral protein expression in microglia, astrocytes, oligodendrocytes and neurons in the spinal cord of TMEV-infected mice**. **A**. Left panes shows microgliosis and astrogliosis (immunocytochemistry, brown cells, red surrounded areas) at sites where viral protein VP1 expression (*in situ *hybridization, silver grains) was present, while right panes shows Iba+ microglia and GFAP+ astrocytes co-localized with VP1 hybridization (orange arrow, higher magnification in inset) **B**. VP1 expression was also found to co-localize with CAII+ oligodendrocytes (top, green arrows) and NeuN+ neurons (bottom, green arrow). Expression of VP1 was not found to occur in TCRαβ-positive T-cells (not shown). In all instances VP1 expression could be found outside of the cell type assayed. Scale bars: green = 5 μm, red = 25 μm.

All of the SJL/J mice injected with the TMEV (7/7) developed a sustained, chronic infection through the month following the intracerebral injection of TMEV. This was exemplified by showing a strong expression of TMEV VP1 protein in their hindbrain and the spinal cord (Fig 1.). The presence of TMEV in the CNS engendered a strong gliosis at the sites of infection (Fig. 2a). With a combination of ISH for VP1 and IC, the cellular loci of infection for the TMEV were revealed to include Iba^+ ^infiltrating macrophage/microglial cells, GFAP^+ ^astrocytes, NeuN^+ ^neurons and CAII^+ ^oligodendrocytes (Fig. 2b). Although TCRαβ^+ ^T-cells were found close to areas of infection, they did not co-localize with VP1 RNA.

The C57BL/6 mice showed much greater susceptibility to the TMEV when receiving the LPS injection regimen (6/10 infected) than only saline injections (1/10 infected). For the sake of consistency, only the C57BL/6 mice on the LPS regimen were included in the study. Comparing VP1 expression, a significant difference was found between SJL/J and C57BL/6 mice (Fig. 1, p < 0.05, F_(1,25) _= 25.85) and TMEV versus vehicle treated mice (p < 0.05, F_(1,25) _= 106.417), as well as a significant treatment × strain interaction (p < 0.05, F_(1,25) _= 28.361). Pair wise comparison revealed significant VP1 expression differences between TMEV and vehicle groups of SJL/J (Bonferroni *t*-test, p < 0.05, t = 9.768) and C57BL/6 (Bonferroni *t*-test, p < 0.05, t = 4.165) mice. Furthermore, the infected SJL/J mice showed a significantly higher VP1 expression than their infected C57BL/6 counterparts (Bonferroni *t*-test, p < 0.05, t = 8.063). However, the demyelination seen in the white mater of spinal cord sections of the two stains was roughly the same (see Figure 1, inset). These results support previous work showing that chronic TMEV-IDD can be attained in the resistant C57BL/6 strain following LPS treatment. However, this infection is not as severe as in the susceptible SJL/J strain.

### Expression of viral component-specific TLRs in TMEV infected SJL/J and C57BL/6 mice

**Figure 3:**
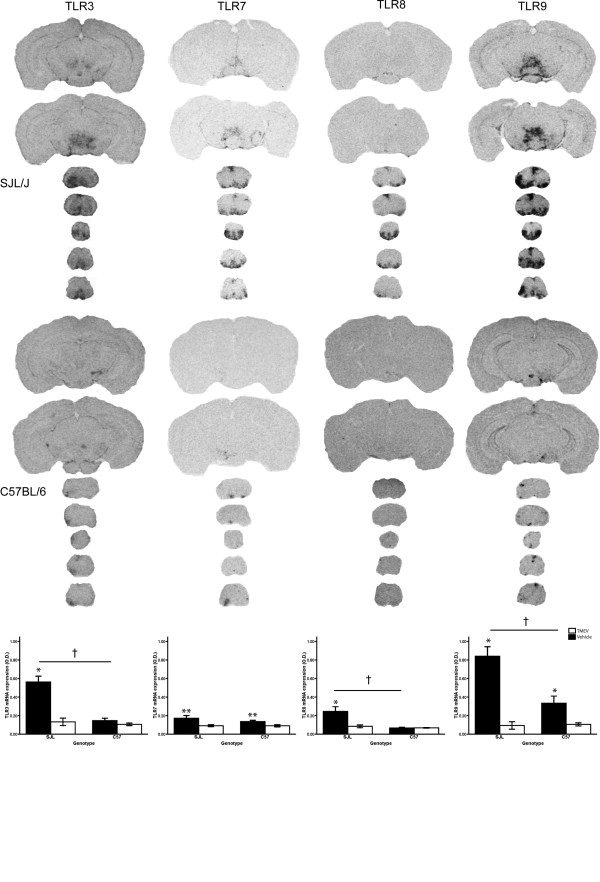
**Expression of Toll-like receptors for viral elements in the central nervous system of SJL/J and C57BL/6 mice after infection with Theiler's murine encephalomyelitis virus**. C57BL/6 mice were further treated twice with lipopolysaccharide to increased susceptibility to Theiler's murine encephalomyelitis virus (TMEV). The coronal sections (25 mm) we taken from X-ray films (Biomax, Kodak, exposed 3 days) and are representative hybridization signals for Toll-like receptors (TLR) TLR3, TLR7, TLR8 and TLR9 near the level of injection (cortical slices) and in the spinal cord. Bottom panel shows quantitative analysis of TLRs signal optical density (O.D.) in representative spinal cord sections. Although comparisons revealed that TLR expression was significantly lower in infected C57BL/6 *versus *SJL/J mice (except TLR7), TLR expression in C57BL/6 was still significantly higher in the TMEV infected *versus *vehicle saline group for TLR7 and TLR9. Data presented as mean ± SEM. **: ANOVA significant main effect of TMEV *vs *Vehicle, p < 0.05; *: significant TMEV *vs *Vehicle pair wise comparison within strain, Bonferonni corrected *t*-test p < 0.05; †: significant SJL/J *vs *C57/BL6 pair wise comparison within TMEV treatment, Bonferonni corrected *t*-test p < 0.05.

The members of TLR receptor family are known for their specificity to bind particular components of infectious agents. In this case, TLR7 specifically recognizes the viral single stranded RNA from TMEV (TLR8 also recognizes ssRNA, but is not thought to be involved in TMEV binding), while TLR3 recognizes its double stranded form that appears during replication. Accordingly, TLR7 expression was found to be significantly upregulated in TMEV infected SJL/J and C57BL/6 mice compared to controls (Fig. 3, p < 0.05, F_(1,25) _= 9.077, no pair wise comparison was done because there was no strain × treatment interaction, p = 0.404, F_(1,25) _= 0.723). However, TLR3 was found significantly upregulated in SJL/J (Bonferroni *t*-test, p < 0.05, t = 6.358), but its expression in C57BL/6 was not significantly increased by TMEV infection (Bonferroni *t*-test, p = 0.431, t = 0.802). TLR8 was also found to be upregulated in SJL/J infected mice (Bonferroni *t*-test, p < 0.05, t = 3.256), but not in C57BL/6 (Bonferroni *t*-test, p = 0.973, t = 0.0340), while TLR9, which recognizes unmethylated CpG motifs in viral and microbial DNA, was found to be significantly upregulated in SJL/J (Bonferroni *t*-test, p < 0.05, t = 6.308) and to a lesser extent in C57BL/6 (Bonferroni *t*-test, p < 0.05, t = 2.576). This TLR expression profile would seem to indicate that TMEV expression can lead to a wide-spread up-regulation of viral component-specific TLRs in the brain, irrespective whether or not they can bind elements of the TMEV. Our results show this is more obvious in a TMEV-susceptible *versus *a TMEV-resistant mouse strain.

### Expression of bacterial component-specific TLRs in TMEV infected SJL/J and C57BL/6 mice

**Figure 4:**
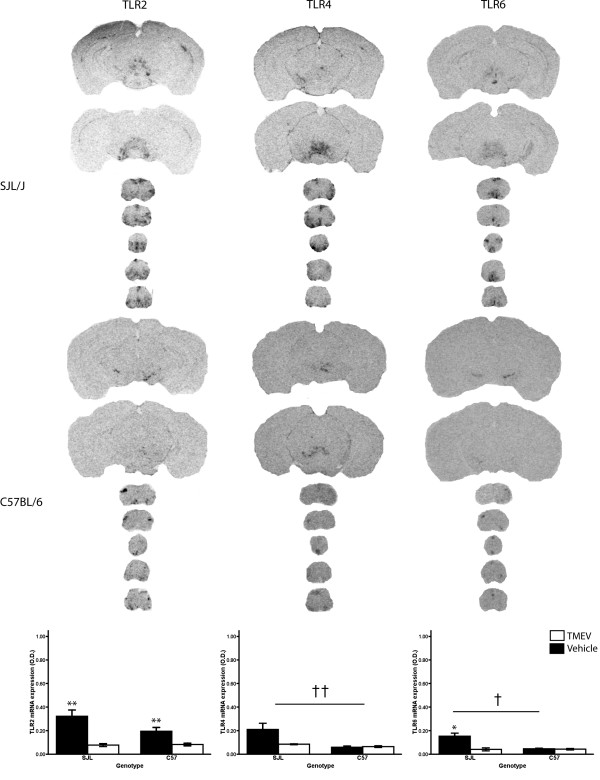
**Expression of Toll-like receptors for bacterial elements in the central nervous system of SJL/J and C57BL/6 mice after infection with Theiler's murine encephalomyelitis virus**. C57BL/6 mice were further treated twice with lipopolysaccharide to increased susceptibility to Theiler's murine encephalomyelitis virus (TMEV). The coronal sections (25 mm) we taken from X-ray films (Biomax, Kodak, exposed 3 days) and are representative hybridization signals for Toll-like receptors (TLR) TLR2, TLR4 and TLR6 near the level of injection (cortical slices) and in the spinal cord. Please note the concordant expression all of the TLRs in areas similar to VP1 expression (see Fig 1) in SJL/J mice. Bottom panel shows quantitative analysis of TLRs signal optical density (O.D.) in representative spinal cord sections. TLR2 and TLR6 were found to be significantly expressed in infected SJL/J mice, while TLR4 expression was noted but did not reach significance. TLR2 was the only TLR expression to reach significance in infected C57BL/6. Vehicle injection had no effect on TLR expression. Data presented as mean ± SEM. **: ANOVA significant main effect of TMEV *vs *Vehicle, p < 0.05; *: significant TMEV *vs *Vehicle pair wise comparison within strain, Bonferonni corrected *t*-test p < 0.05; ††: ANOVA significant main effect of SJL/J *vs *C57BL/6, p < 0.05; †: significant SJL/J *vs *C57/BL6 pair wise comparison within TMEV treatment, Bonferonni corrected *t*-test p < 0.05.

The TMEV-infected mice also show an up-regulation in bacteria-associated TLR mRNA. Both strains exhibit an increase in TLR2 expression (Fig. 4, p < 0.05, F_(1,25) _= 23.492, no pair wise comparison was done because there was no strain × treatment interaction, p = 0.084, F_(1,25) _= 3.284). Although TLR2 is the main receptor for components of the cell wall of Gram positive bacteria, its expression is also associated with microglial activation. The TLR6 mRNA expression was only upregulated by the TMEV infection in SJL/J (Bonferroni *t*-test, p < 0.05, t = 4.224). The Gram negative bacteria-associated TLR4 showed visually but insignificant increases in expression (p = 0.074, F_(1,25) _= 3.529). TLR4 is recognized as being expressed constitutively and not regulated even by its own ligand, LPS.

## Discussion

To establish a persistent autoimmune state of demyelination in the mouse, the TMEV infection must involve a multitude of viral-host interactions that increase the reactivity of the host immune system in the CNS to the point that a host response to myelin elements occurs. It is well established that TLRs recognize components of viruses and are involved with the initiation of the first line of defense against foreign particles. It is now emerging that TLRs can also recognize and bind endogenous proteins, and their presence (or absence) is discussed and often implicated in various model of immune diseases such as lupus, arthritis and MS (see Ehlers and Ravetch[13] and Papadimitraki, Bertsias and Boumpas[6] for reviews). The implication of TLRs in the genesis of TMEV-IDD seems even more relevant when it comes to establishing this model in the resistant C57BL/6 mice, where LPS treatment, a TLR4 ligand, is needed to stimulate the chronic autoimmune state in the mice. The endotoxin LPS is known to up-regulate multiple TLRs in the mouse brain and in TMEV-infected microglia[3,4]. This could support the direct or indirect (by the induction of cytokines and chemokines) involvement of TLRs in the initiation of TMEV-IDD in C57BL/6 resistant mice following LPS stimulation.

The involvement of TLRs in the TMEV-IDD is well supported by the results of the current experiments: multiple TLRs are up-regulated by the TMEV in susceptible SJL/J as well as in the resistant C57BL/6 treated with LPS. These TLRs include TLR7, the cognate receptor for TMEV, TLR9, and TLR2. The infected SJL/J mice also show a significant up regulation of TLR3, TLR6 and TLR8. The expression of these receptors is observed in C57BL/6, but does not reach significance. The expression of TLR4 is seen in both strains, but does not reach significance due to higher baseline expression and variability. A similar expression of TLRs upregulated by TMEV was reported earlier, but focused on the *in vitro *induction by TMEV in cultured microglia[4]. The use of our *in vivo *approach permits us to observe the co-localization of the TLR expression to the specific areas of the CNS where the TMEV was detected. This method also confirms that the TMEV is able to infect all of the local cell populations of the CNS. Yamada and colleagues had previously reported similar spatial and temporal viral distribution[14].

Both the susceptible strain and the resistant stain stimulated with LPS are able to up-regulate TLRs following TMEV-IDD, with obvious differences, namely the intensity of the TLR expression and the lack of significant TLR3, 6 and 8 up-regulation in C57BL/6 mice. TLR6 and 8 are not commonly associated with autoimmune or demyelinating processes, so their presence in the SJL/J mice could be a simple consequence of the greater immune response taking place in the SJL/J. On the other end, the absence of a significant TLR3 upregulation and low TLR7 and 9 up-regulation compared to the susceptible mice the infection could hint at a possible mechanism by which LPS could enhance their response to the TMEV. The expression of these TLRs is in CNS regions where TMEV abounds, thus it could reflect the higher viral titer in the SJL/J mice. That would appear to be central in the enhanced response by the susceptible mice is the intensity of the TLR signal. By having a higher number of TLRs on the surface and inside the endosomes and lysosomes of the immune cells, it could increase their susceptibility to inadvertently recognize self antigens and precipitate an autoimmune reaction. The chance of an autoimmune response could be potentiated by any cell death (oligodendrocytes, neurons, microglia, etc.) occurring at the loci of infection and demyelination. The nucleotides released by the dying cells could bind the nucleotide-specific TLRs (TLR3, 7 and 9) and trigger a self response. Even if these receptors are located in the endosomes and are hard to reach, this has already been discussed for other autoimmune diseases such as lupus[15] and could also be at the root of the importance of the LPS stimulation in the resistant mice. This phenomenon especially pertains to plasmacytoid dendritic cells (pDCs) that could make their way into the CNS from the periphery. This specific class of dendritic cell expresses high levels TLR3, TLR7 and TLR9, which were all upregulated in the CNS of the TMEV-infected susceptible mice (for review, see Gilliet et al[16]). A possible mechanism by which the pDCs could be involved in autoimmune process is the recognition of self DNA (from dying cells) aggregates by TLR9. These aggregates are possible through the interaction of self DNA and high-mobility group box 1 protein with LL37 (both released from dying cells). This complex could be delivered by lipid rafts or TLR9-containing endosomes in pDCs and trigger an autoimmune response[16]. Although the expression of TLR3, 7 and 9 is lower in the resistant C57BL/6 mice, LPS is known to up-regulate their expression in immune cells [4,17]. Even if this up-regulation is transient, and thus not detected several weeks after in LPS injection in the resistant mice, it could be enough to activate peripheral macrophages and pDCs and precipitate the autoimmune phenomenon described above. This early activation by LPS could compensate for a weaker response of the pDCs in the resistant strain of mice. Interestingly, pDCs are one of the main therapeutic targets for MS[16], and thus the involvement of TLRs in the processing of self-DNA in these cells could be a major player in the treatment of the disease.

The mechanism by which TLRs could play a role in autoimmunity is still debated. Some have suggested that Th1-mediated autoimmunity could be mediated through MyD88 activation, a common signal transduction protein used by most TLRs[18]. Furthermore, TLR9 and MyD88 signaling have been linked to class switching to pathogenic IgG2a and 2b auto-antibodies in lupus[19]. Thus, modulation of that action of TLRs and MyD88 could provide potential therapeutic targets for the treatment of immune disorders. Early warning signs of immune disorders could also be deciphered by looking at the reactivity of the signaling through TLRs, which could open the door to detecting such disorders promptly and optimizing their treatment at an earlier phase in their development.

## Conclusion

This study clearly describes the presence of multiple TLRs in the brain of mice during TMEV-IDD. These include TLRs involved in the recognition of the viral genome (TLR3 and TLR7) and multiple other TLRs that could be involved in autoimmune processes. The direct link between TLRs and the autoimmune and other mechanisms involved in the establishment of the chronic deregulated immune state observed during TMEV-IDD remains to be elucidated, but reinforces TLRs as possible culprits in the initiation of self-immune processes in the brain. This could encourage the investigation of TLRs as possible therapeutic targets in autoimmune/demyelinating diseases.

## Competing interests

The author declares that they have no competing interests.
